# Analysis of HR-HPV Infection Concordance Rates in Cervical and Urine Specimens; Proposal of Additional Cervical Screening Process for Women Who Refuse Invasive Cervical Sampling

**DOI:** 10.3390/jpm12121949

**Published:** 2022-11-24

**Authors:** Dong Hyeok Kim, Hyunwoo Jin, Kyung Eun Lee

**Affiliations:** 1 Department of Clinical Laboratory Science, College of Health Sciences, Catholic University of Pusan, Busan 46252, Republic of Korea; 2 Clinical Trial Specialist Program for In Vitro Diagnostics, Brain Busan 21 Plus Program, The Graduate School, Catholic University of Pusan, Busan 46252, Republic of Korea

**Keywords:** human papillomavirus, cervical cancer, urine, real-time PCR

## Abstract

This study aimed to provide basic data for the clinical application of urine samples to prevent cervical cancer due to persistent HR-HPV infection in women who refuse invasive cervical sampling. Pairs of cervical swabs and urine samples were collected from 210 asymptomatic women who visited the obstetrics and gynecology department from August to December 2020, and a total of 420 samples were collected. Using the PANA RealTyper™ HPV Screening Kit as a real-time PCR method, paired cervical swabs and random urine samples were tested. A total of 19 samples (9.1%) were both HPV positive and 177 (84.3%) were both negative. The concordance between the two types of samples was 93.3%, with κ = 0.69 (moderate, 95% CI 0.54–0.84). The HPV infection rate by age was highest in both cervical swabs and urine samples in women in their 30s, followed by those in their 20s. Thus, the HPV infection rate was high in young women under 40 at 69.2% in cervical swabs and 61.8% in urine samples. Urine samples are considered a valuable screening test for women who refuse invasive Pap tests to prevent cervical cancer caused by persistent HPV infection.

## 1. Introduction

Cervical cancer incidence in many developed countries has decreased significantly with the introduction of Pap test based cervical cancer screening programs, emphasizing the importance of early diagnosis and prevention [1]. However, the rate of HPV infection, which is known to be the main cause of cervical cancer, is reportedly increasing among young women. Ferris et al. (2020) reported that women aged 24–34 years (20.0%) showed higher prevalence of nine types of HPV than women aged 34–45 years (12.7%) in seven countries (the United States, France, Germany, Spain, Colombia, the Philippines, and Thailand) [2]. In Korea, a high HPV infection rate among young women aged 18 to 29 years was also reported at 49.9% [3]. Therefore, to prevent cervical cancer onset in young women, early diagnosis and treatment methods are required to prevent continuous HPV infection.

In Korea, in order to prevent the increase in cervical cancer incidence and the rate of HPV infection among people in their 20s and 30s, a screening system has been established that allows women over 20 to receive a Pap test every two years for free [3,4]. However, according to the Korean National Cancer Screening Survey in Korea in 2018, the screening rate for cervical cancer for women in their 20s was 20.9%, significantly lower than that of other age groups [5]. This was attributed to psychological reluctance, where unmarried women would find it awkward to visit an obstetrician, and the invasive Pap test would be uncomfortable and painful [6,7]. In order to increase the participation rate for cervical cancer screening among young women, the needs of young women should be accommodated through the development of non-invasive screening methods, as well as efforts to change social awareness.

Arbyn et al. (2014); Arbyn et al. (2018) recommended HPV testing using self-collected specimens as a strategy to increase the screening rate in women who do not undergo regular cervical cancer screening [8,9]. However, this carries a high possibility of contamination due to the lack of experience in sample collection and sealing after collection, and the cumbersome procedure for sending by mail was not very effective [10,11]. On the other hand, in patients with high-grade intracervical lesions, HPV detection concordance rates between the cervical and urine specimens were 79.8% and 86.2%, respectively, indicating relatively meaningful results for usefulness [12,13]. Urine specimens are considered very useful, because they are easy and simple to collect in a non-invasive way. However, since this method detects HPV through exfoliated cervical cells, rather than the actual HPV infection site, it is estimated that HPV DNA may be destroyed by urine inhibitors, necessitating additional research [14,15,16]. Pathak et al. reported that urine detection of high-risk HPV showed sensitivity and specificity of 77% and 88%, respectively. In particular, urine detection of HPV 16 and 18 showed sensitivity and specificity of 73% and 98%, respectively [17]. Therefore, urine sampling can provide an effective alternative in the age group where cervical cancer screening is not performed properly.

The purpose of this study was to confirm the usefulness of using urine samples as a screening test to prevent cervical cancer caused by persistent HR-HPV infection in women who refused invasive cervical sampling and to suggest an additional cervical screening process.

## 2. Materials and Methods

### 2.1. Participants

A pair of cervical swabs and urine samples were collected from 210 asymptomatic women who visited the obstetrics and gynecology department from August to December 2020, and a total of 420 samples were collected. Cervical swab samples from patients who visited the obstetrics and gynecology department were collected by trained gynecologists, and urine samples were self-collected. This study was conducted under the approval of the Institutional Ethics Committee of the Catholic University of Busan (approval number CUPIRB-2020-01-011).

### 2.2. DNA Extraction

DNA extraction was performed using 5% Chelex^®^ 100 Resin solution (Bio-Rad, Hercules, CA, USA) according to the manufacturer’s protocol. 200 μL of the sample was transferred to a new E-tube, centrifuged at 8000× *g* for 10 min, the supernatant was discarded, and 100 μL of 5% Chelex^®^ 100 Resin solution was added to the remaining cell pellet, followed by vortexing and spinning down. Then, samples were incubated at 100 °C for 10 min, centrifuged at 11,000× *g* for 10 min, and supernatant transferred to a new E-tube. The extracted genomic DNA was checked for concentration and purity using a NanoDrop 2000 (Thermo Fisher Scientific, Wilmington, DE, USA) and stored at −18 °C before analysis.

### 2.3. HPV Detection Using Real-Time PCR in Pairs of Cervical Swab and Urine Samples

The PANA RealTyper™ HPV Screening Kit (PANAGENE, Daejeon, Republic of Korea) detected HPV DNA through real-time PCR (qPCR) using CFX96 (Bio-Rad, Hercules, CA, USA) for 14 high-risk (16, 18, 31, 33, 35, 39, 45, 51, 52, 56, 58, 59, 66, 68) and two low-risk (6, 11) HPV subjects. For real-time PCR, 5 μL of DNA extracted from the sample, 19 μL of HPV mix, and 1 μL of Taq DNA polymerase were mixed to a total of 25 μL. Then, by setting a baseline for each fluorescent dye, infection and HPV genotype were confirmed according to the melt temperature value of the melting peak for each fluorescent signal.

### 2.4. HPV DNA Sequencing

For HPV DNA sequencing, primary amplification was performed using *MY11/MY09* primers, and nested PCR was performed for secondary amplification using *GP5/GP6* primers [18]. The nucleotide sequence of *MY11* is GCM CAG GGW CTA TAA YAA TGG, *MY09* is CGT CCM ARR GGA WAC TGA TC, *GP5* is TTT GTT ACW GTK GTR GAT AC, and *GP6* is GAA AAA TAA ACT GTA AAT CAT ATT C. Each PCR product was analyzed by electrophoresis in 2% agarose gel. HPV-infected specimens were detected at 150 bp. Gels near 150 bp were extracted for HPV DNA sequencing. PCR products were re-amplified using BigDye Terminator (Applied Biosystems, Waltham, CA, USA) and GP5 primers, and HPV DNA sequencing was performed using ABI3730XL (Applied Biosystems, Waltham, CA, USA). The Basic Local Alignment Search Tool version 2.10.1 (https://blast.ncbi.nlm.nih.gov/, accessed on 8 August 2020) was used for HPV genotyping.

### 2.5. Data Analysis

Concordance between the results was analyzed using Kappa statistics (Cohen’s Kappa, κ) and defined as “None” (0.00 ≤ κ ≤ 0.19), “Minimal” (0.20 ≤ κ ≤ 0.39), “Weak” (0.40 ≤ κ ≤ 0.59), “Moderate” (0.60 ≤ κ ≤ 0.79), “Strong” (0.80 ≤ κ ≤ 0.89), “Almost perfect” (0.90 ≤ κ < 1), or “perfect” (=1) [19].

## 3. Results

### 3.1. Study Population Characteristics

A total of 420 cervical swabs (n = 210) and random urine samples (n = 210) were collected from 210 women aged 20 to 85 years (mean age 39.9 years) and used in this study. Of all 210 women, the age distribution included 42 (20.0%) women in their 20s, 81 (38.6%) in their 30s, 42 (20.0%) in their 40s, 25 (11.9%) in their 50s, and 20 (9.5%) in their 60s and older.

### 3.2. HPV Detection Results

#### 3.2.1. HPV DNA Detection Using Real-Time PCR in Pairs of Cervical Swabs and Urine Samples

Using the PANA RealTyper™ HPV Screening Kit as a real-time PCR method, 19 (9.1%, 19/210) cervical swabs and random urine samples were HPV-positive, and 177 samples (84.3%, 177/210) were negative. The concordance between the two types of samples was 93.3%, with κ = 0.69 (Moderate, 95% CI 0.54–0.84). The prevalence of HR-HPV positivity for 14 types, including HPV 6 and 11, was the same at 12.4% (26/210) in cervical swabs and urine samples (Table 1).

#### 3.2.2. HPV Positive Rates and Age Distribution in Cervical Swabs and Random Urine Samples

HPV positivity in cervical swabs was 30.8% (8/26), 38.4% (10/26), 11.5% (3/26), 11.5% (3/26), and 7.8% (2/26) in under 20, 30, 40, 50, and over 60 years of age, respectively. HPV positivity in random urine samples was 26.9% (7/26), 34.6% (9/26), 19.2% (5/26), 15.4% (4/268), and 3.9% (1/26) in under 20, 30, 40, 50, and over 60 years of age, respectively. The HPV infection rate by age was highest in both cervical swabs and urine samples for women in their 30s, followed by those in their 20s. That is, in young women under 40, HPV infection rate was high at 69.2% (18/26) in cervical swabs and 61.8% (16/26) in urine samples (Table 2).

#### 3.2.3. Analysis of HPV DNA Sequencing Detection According to HPV Real-Time PCR Results

In real-time PCR analysis, in the case of cervical swabs/urine (+/+), HPV DNA sequence was detected in 78.9% (15/19) of cervical swabs and 68.4% (13/19) in urine. In the case of cervical swab/urine (+/−), the cervical swab sequencing detection rate was 57.1% (4/7), which was higher than the urine sequencing detection rate of 14.3% (1/7). On the other hand, in the case of cervical swab/urine (−/+), the detection rate for cervical swab sequencing was 28.6% (2/7), which was lower than the urine sequencing detection rate of 57.1% (4/7). In the case of cervical swab/urine (−/−), the detection rates of cervical swab and urine sequencing were the same at 2.8% (5/177) (Table 3).

## 4. Discussion

Persistent HPV infection is known as a major cause of cervical cancer [20]. However, up to 80% of cervical cancer cases are preventable through regular screening and early diagnosis [21]. Therefore, persistent HPV infection is considered to increase the risk of developing cervical cancer in patients not participating in cervical cancer screening. However, there are limitations in the screening system for preventing cervical cancer because there are many women who reject the current cervical Pap test [14,15,16]. For non-participants who are reluctant to have both cervical sampling and vaginal self-sampling, HPV testing using urine samples has proven its value in various studies, because it is non-invasive and easy to collect [12,13,17,22].

In another study comparing HPV detection in cervix and urine samples from patients with high-grade lesions of the cervix, the concordance rates of the two types of samples were 79.8% (κ = 0.36) and 86.2% (κ = 0.72) [12,13], respectively. In this study, HPV test results using real-time PCR (concordance = 93.3%, κ = 0.69, 95% CI 0.54–0.84) for collected cervical swabs and urine samples showed a moderate (0.60 ≤ κ ≤ 0.79) concordance rate. In addition, in another study in Korea including 101 patients diagnosed with a cervical high grade squamous intraepithelial lesion and ovarian disease, HPV detection in cervical and urine samples was compared using three kits; RealTime HR-S HPV (Sejong Medical Co., Ltd., Paju, Republic of Korea), Anyplex^TM^ II HPV 28 (Seegene, Seoul, Republic of Korea), and Cobas 4800 HPV (Roche Molecular Diagnostics, Pleasanton, CA, USA), which showed concordance rates of 84.2% (κ = 0.51), 81.2% (κ = 0.44) and 86.1% (κ = 0.33), respectively [23]. Although most of the women in this study were asymptomatic and healthy, the concordance rate between the cervical swabs and urine samples were higher than in previous studies, showing statistical significance. Although the diagnostic method was different in the previous study, the concordance rate of HPV detection between the cervical and urine samples were similar to that in our study [24,25]. An E6/E7 DNA qPCR test showed a concordance rate of 84.8% [24], and HPV REBA showed a concordance rate of 94.44% [25]. Although there are differences depending on the experimental technique and the type of diagnostic kit, HPV concordance rates were similar for cervical swabs and urine samples.

Recently, a high rate of HPV infection in women in their 20s has been reported in Korea [3]. We detected a 19.0% (8/42) infection rate in cervical specimens from women in their 20s, as well as 16.7% (7/42) in urine specimens, higher than that of other age groups. This is because HPV is a very common sexually transmitted virus in young people within the first few years of sexual activity [26]. Although not all HPV infections lead to cervical cancer, persistent infection is closely related to its development [27]. Therefore, continuous HPV infection is likely to cause female genital diseases in young women with a low cervical cancer screening participation rate.

**Figure 1:**
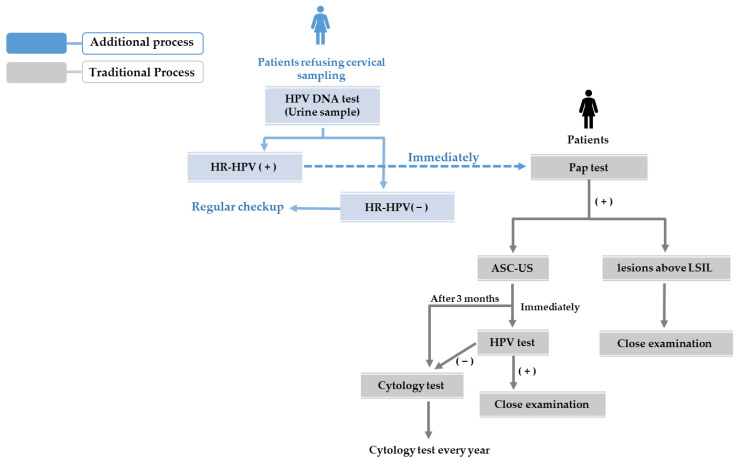
Proposal of additional cervical screening process for the prevention of HR-HPV infection in women who refuse invasive cervical sampling. ASC-US; atypical squamous cells of undetermined significance, LSIL; low grade squamous intraepithelial lesion.

In this study, HPV detection results using real-time PCR were 12.4% (26/210) in the cervix and urine samples, lower than the HPV prevalence demonstrated in other studies [12,13,23]. This is explained by the fact that all women who participated in the general examination were in a healthy condition without symptoms, and the diagnostic kit only detects high-risk types of HPV 14, including types 6 and 11. In addition, since urine samples do not directly detect HPV, evaluating infection through cervical ectopic cells, inaccuracy can be suspected, because there are not enough cervical cells [14,28]. Therefore, in this study, we sequenced HPV DNA after performing nested PCR to confirm HPV infection by constructing primers targeting the *MY11/MY09* and *GP5/GP6* genes. When both a cervical swab and urine were positive for HPV real-time PCR, HPV DNA sequencing was detected in 15 out of 19 cases (78.9%) in cervical swab, and 13 cases (68.4%) in urine samples. The sequence was detected. Also, out of 177 cases where both cervical swab and urine were negative in HPV real-time PCR, HPV DNA sequencing was detected in five cases (2.8%) each of cervical swabs and urine samples. In this study, nested PCR is an HPV DNA sequencing method with high sensitivity and specificity, but it can detect only a single infection, limiting the accuracy of determining HPV complex infections. However, the results of this study suggested that HPV could also be detected in urine samples. In this study, an experiment was conducted on random urine from asymptomatic women. Various studies on urine samples, such as first-void urine, first urine in the morning, daily urine, and self-collected samples, should be continued to standardize the type, collection method, and storage of urine samples that are advantageous for HPV detection [28,29]. In addition, in order to increase the screening rate for patients who refuse invasive cervical screening, especially young women, and to prevent the occurrence of cervical tumors, it is necessary to specifically explore the use of urine samples. As shown in Figure 1 [30], if the process of adding HPV DNA testing using urine samples to the national screening for cervical cancer is introduced, it is thought that it will be helpful for healthy management of female reproductive organs.

## 5. Conclusions

In this study, the concordance of HPV infection between cervical swabs and urine samples was found to be 93.3%. HPV DNA testing using urine samples is evaluated as a valuable screening test for cervical cancer prevention for women who refuse the invasive pap test. In addition, the introduction of an additional cervical screening process for clinical application of urine specimens is expected to help prevent cervical cancer caused by HPV infection by increasing the screening rate of women.

## Figures and Tables

**Table 1:** Comparison of HPV detection rates in cervical swabs and random urine samples from 210 women.

Sample		Urine		Total	Concordance Rate	Cohen’s Kappa (*κ*) *
		Positive, *n* (%)	Negative, *n* (%)			
Cervical swab	Positive	19 (9.1)	7 (3.3)	26 (12.4)	93.3%	0.69 (95% CI 0.54–0.84)
	Negative	7 (3.3)	177 (84.3)	184 (87.6)		
	Total	26 (12.4)	184 (87.6)	210 (100)		

* Cohen’s Kappa, κ: “None” (0.00 ≤ κ ≤ 0.19), “Minimal” (0.20 ≤ κ ≤ 0.39), “Weak” (0.40 ≤ κ ≤ 0.59), “Moderate” (0.60 ≤ κ ≤ 0.79), “Strong” (0.80 ≤ κ ≤ 0.89), “Almost perfect” (0.90 ≤ κ < 1), or “perfect” (=1).

**Table 2:** Comparison of HPV positive rate by age between cervical and urine samples.

Age	Cases, *n* (%)	HPV Positive	
		Cervical Swab, *n* (%)	Urine, *n* (%)
≤29	42 (20.0)	8 (30.8)	7 (26.9)
30–39	81 (38.6)	10 (38.4)	9 (34.6)
40–49	42 (20.0)	3 (11.5)	5 (19.2)
50–59	25 (11.9)	3 (11.5)	4 (15.4)
≥60	20 (9.5)	2 (7.8)	1 (3.9)
Total	210 (100)	26 (100.0)	26 (100.0)

**Table 3:** Analysis of HPV DNA sequencing detection according to HPV real-time PCR results.

Real-Time PCR Results	Sequencing Analysis	
	Cervical Swab Detection, *n* (%)	Urine Detection, *n* (%)
Cervical/Urine (+/+) *n* = 19	15 (78.9)	13 (68.4)
Cervical/Urine (+/−) *n* = 7	4 (57.1)	1 (14.3)
Cervical/Urine (−/+) *n* = 7	2 (28.6)	4 (57.1)
Cervical/Urine (−/−) *n* = 177	5 (2.8)	5 (2.8)
